# The Circulation in the Kidney

**Published:** 1888-05

**Authors:** Bat. Smith

**Affiliations:** Wharton, Texas


DANIEL'S
### Texas Medical Journal.

Published Monthly.—Subscription $2.00 a Year.

Vol. 3. AUSTIN, MAY, 1888. No. 11.

By unanimous vote of the members, this Journal was made the Official Organ of the Austin District Medical Society.

### Original Articles.

☞CONTRIBUTED EXCLUSIVELY TO THIS JOURNAL.☜

*The Articles in this Department are accepted and published with the understanding that we are not responsible for, nor do we indorse the views and opinions of the writers by so doing.*

### CIRCULATION IN THE KIDNEY.

*By Bat. Smith, M. D., Wharton, Texas.*

For Daniel's Texas Medical Journal.

I hardly expect that the statements which I here make will be accepted by the profession without some degree of skepticism, yet I am convinced that, if those versed in histology will give the subject their careful consideration, and examine the structure of a healthy kidney with minute care, they will become convinced of the error of our earlier anatomists. For many years we have held the circulation in the kidney to be substantially as follows:

The malpighian bodies, found only in the cortical substance of the kidney, are small, round bodies, of a deep red color, averaging in diameter 1-120 of an inch. Each body is composed of a vascular tuft, inclosed in a thin membranous capsule. The vascular tuft consists of the ramifications of a minute artery, the afferent vessel, which, after piercing the capsule, divides in a radiated manner into several branches, which ultimately terminate in a finer set of capillaries. From these a small vein, the efferent vessel, proceeds; piercing the capsule near the afferent vessel, it forms, with other efferent vessels, a close plexus around neighboring tubuli, called the tubular plexus. The renal artery divides into four or five branches which enter the hilus of the kidney, being invested by sheaths derived from the fibrous capsule. They penetrate the substance of the organ between the papillae, and enter the cortical substance in the intervals between the medullary cones, dividing and subdividing in their course towards the bases of the pyramids, forming arches by their anastomoses. From these arches numerous vessels are distributed to the cortical substance, some of which, the afferent vessels, enter the malpighian corpuscles, whilst others form a network of capillaries around the uriniferous tubules.

The veins commencing upon the surface of the organ pass inward, opening into larger veins around the bases of the medullary cones; after receiving the plexus from the tubular portion they accompany the branches of the arteries to the sinus of the kidney, finally uniting to form a single vein, which terminates in the inferior Vena Cava. That this view of the anatomy of the kidney, as far as its circulation is concerned, should have been accepted by histologists as the correct one without dispute, is not at all strange, considering that long before the microscope had reached its present degree of perfection this supposed fact was looked upon as sufficiently well established not to warrant investigators wasting their time over a subject acknowledged by them as already settled.

The afferent vessel is a small artery, possessing all the characters of vessels of that class; the so-called capillaries around the Malpighian bodies, however, are *not* capillaries at all, but *small arteries*. We have only to compare the structure of an arteriole to that of a capillary. The walls of a capillary vessel consist of a fine, transparent, homogeneous membrane, in which are imbedded, at intervals, minute oval corpuscles, the remains of the nuclei of the cells from which the vessel was originally formed. In the largest capillaries we sometimes find a trace of an epithelial lining, and a few filaments circularly dispersed.

Now, the structure of these so-called capillaries differs widely from that just described. We find, upon close examination, the internal coat of these vessels to consist of two distinct layers, viz: A single layer of fusiform shaped epithelial cells, generally with round, and sometimes oval nuclei, and a transparent, colorless, shining membrane, perforated with small elongated apertures, the well known fenestrated membrane of Henle. The elastic layer in these arterioles is very delicate, but the epithelium can be plainly seen.

The so-called efferent vessel is not a vein, but an arteriole possessing the same structure and character as the afferent vessel. The network of capillaries found roundthe uriniferous tubules are true capillaries, their structure differing materially from that of the vessels in the first network belonging to the Malpighian bodies.

If we consider the large size of the renal artery and the small size of the organ which it supplies, together with the large amount of blood carried to the kidneys, (over two thousand pounds in twenty-four hours), we may be able to account for this seeming anomally in the circulation of the kidney. The renal arteries arise from the sides of the Aorta, just below the superior Mesenteric artery, and are directed outward, forming nearly a right angle with the Aorta, and enter the kidney after having been divided into four or five branches. Now, with every contraction of the left ventricle a pressure of two and one half to nearly three pounds is exerted. In the descending Aorta about the origin of the renal arteries, the pressure is from one and seven eighths to two and one fourths pounds. The renal arteries are very short, and of large calibre, and for that reason they are subdivided into the four or five branches, as mentioned above, before entering the kidney, thereby diminishing the pressure. This, however, is not sufficient, for such a pressure would prove too great for vessels of so weak a structure as those of the capillary system, consequently the afferent vessels break up into a network of smaller arteries, relieving the pressure by offering a larger surface to the volume of blood, and thereby retarding its flow.

In order to make a thorough and satisfactory examination the section should be cut as thin as possible, with a very sharp razor. After placing the specimen on the slide, a drop or two of water should be added. The object glass used in these examinations should have a rather high angular aperture. The investigator will derive much more satisfaction if, instead of using a mirror in work of this kind, he will use the prism, the latter bringing the object out in bold relief, while the mirror causes only a flat picture to be presented.

---

NOTE 1.—This subject was first called to my attention by my former preceptor, Dr. H. D. Schmidt, of New Orleans, formerly Professor of Physiology at the New Orleans School of Medicine, at present Pathologist of the Charity Hospital, New Orleans, La.

NOTE 2.—In the axilla of the common Sloth, (Bradypus tridactylus,) we find a somewhat similar arrangement of the blood vessels, viz: The axillary artery breaks up into a large number of small arteries, which anastomosing with each other, eventually come together to form the brachial artery.

May 5, 1888.

